# "*I was in the darkness but the group brought me light*": Development, relevance and feasibility of the Sondela HIV adjustment and coping intervention

**DOI:** 10.1371/journal.pone.0178135

**Published:** 2017-06-01

**Authors:** Nwabisa Shai, Yandisa Sikweyiya, Ingrid van der Heijden, Naeemah Abrahams, Rachel Jewkes

**Affiliations:** 1 Gender and Health Research Unit, Medical Research Council, Pretoria, South Africa; 2 School of Public Health, University of the Witwatersrand, Johannesburg, South Africa; 3 School of Public Health and Family Medicine, University of Cape Town, Cape Town, South Africa; 4 School of Public Health, University of Western Cape, Bellville, South Africa; 5 School of Health and Rehabilitative Sciences, Faculty of Health, University of Cape Town, Cape Town, South Africa; Stellenbosch University, SOUTH AFRICA

## Abstract

Developing interventions that address psychosocial wellbeing of people living with HIV is critical to ensure strong linkages to and retention in HIV care. This paper describes the development of Sondela, an HIV adjustment and coping intervention for heterosexual men and women living with HIV, and its relevance and feasibility in the South African context. Sondela is a six three-hour, small group-based, participatory workshop series. We followed an iterative, multi-phased process of curriculum development that involved research, theoretical frameworks and piloting. A systematic review highlighted the absence of psychosocial interventions targeting heterosexual HIV positive populations living in high HIV prevalence and resource-poor settings. Formative studies demonstrated risk and social factors associated with adjustment and coping with HIV, emphasising the need for interventions that acknowledge gendered experiences. Our pilot of Sondela demonstrated high levels of relevance and feasibility. Men appreciated the workshop “space” to openly talk about their HIV positive status and what is means for their role as partners and fathers and friends. Women valued the skills and approaches because they were relevant to “real life” situations and not just about HIV. Sondela promises to be valuable in supporting health system initiatives and psychosocial support to strengthen linkages to and retention in HIV care, and this suggests a need for rigorous evaluation of Sondela to establish evidence for its effectiveness in a general population.

## Background

HIV infection imposes a significant psychological burden on men and women. People living with HIV (PLWH) often experience mental health problems such as depression and anxiety after their diagnosis [1]. These are commonly linked to HIV related stigma, problems with disclosure [2], and loss of social support [3–5]. Whilst men experience more self-stigmatisation than women [6], women report more external stigmatisation including disclosure-related partner violence [7, 8]. Higher sexual relationship power among HIV positive women is significantly associated with lower depression and better mental health status compared with women with lower power [9]. Internalized notions of masculinity that men use to negotiate social power and status undermine men’s health [10]. This highlights the need to acknowledge the impact of gender and relationship dynamics on men and women’s ability to cope and adjust to HIV infection.

South Africa has one of the largest HIV epidemics in the world. In 2015, an estimated 7 million people were living with HIV of which 380,000 were new infections, 48% of HIV positive adults and 95% of HIV positive pregnant women received ART, and there were 180,000 AIDS-related deaths in the same year. In sub-Saharan Africa 62% of PLWH know their HIV positive status [11]. Increased coverage of HIV testing and counselling (HCT), and early initiation of ART more recently, have been the primary focus of the South African government response. Increased access to treatment and care ensures improved clinical outcomes and minimize the development of resistance to ART [12]. However the effectiveness of such HIV programmes is undermined by the high proportion of loss to follow up between HCT and ART initiation [13], and the low average CD4 levels at which patients are enrolled on to ART [14]. Initially the South Africa Government (SAG) initiated ART from a CD4 threshold of 250 cells/mm^3^ or less before adopting the World Health Organization (WHO) recommendations of ART initiation at 350 to 500 cells/mm^3^ [15]. In May 2016 the SAG announced the commencement of the test and treat approach from September 2016, to ensure and improve retention throughout the HIV treatment cascade.

Given the large scale rollout of ART, the concomitant responsibility to ensure adherence is immense. Ensuring optimal adherence and improvement to the quality of life of people living with HIV is therefore of great concern [16–18]. Higher levels of early retention in HIV care are associated with adherence, particularly higher levels of viral load suppression and lower cumulative viral load burden [19]. Yet systematic reviews indicate common retention patterns in pre-ART care with substantial loss of patients at every step of the treatment cascade, starting with patients who do not return for their initial CD4 count results and ending with those who do not initiate ART despite eligibility [20]. In South Africa there is limited empirical evidence on how well people cope with and adjust to living with HIV, both pre-ART and on ART. Consequently, there is need to ensure sustained linkages to care and its increased impact on coping and adjustment.

Linkages to and retention in HIV care can be assessed through CD4 testing and receiving the results, ART eligibility assessment and enrolment in South Africa. National estimates indicate 51.7% of PLWH are linked to care with provincial variation between 45.1% to 71.6% [21]. Studies have found disparities that impact on linkages to and retention in HIV care [14, 22–26]. Some of the gaps in linkages to and retention in care are due to individual, social, environmental and health systems factors. Being younger and male are associated with higher levels of attrition [23, 27–29]. Disbelieving HIV results, limited time to seek health care, believing that ART can make you sick and drinking alcohol predict weaker linkages to care [30]. Similarly, perceived ART side effects, perception of HIV as a stigmatizing disease, and non-disclosure to partner decreased linkages to and retention to care [31]. Health system factors included variation between facilities in measures of linkage, and in quality of pre- and post-ART care [24]. Among factors associated with stronger linkages to and retention in HIV care were HIV disclosure [27], being clinically symptomatic including having a lower CD4 count and TB symptoms [27], and believing that ART drugs are available at health facilities [32]. Addressing the barriers and enhancing factors that enable coping and adjustment to positive HIV status is essential to improve linkages to care. This can be done through standardized and theoretically framed interventions.

This paper describes the development of Sondela, an HIV adjustment and coping intervention for adult heterosexual men and women recently diagnosed with HIV, and its relevance to participants during the pilot phase and feasibility in the context of South Africa.

### The Sondela intervention

Sondela is an HIV coping and adjustment intervention designed to address the immediate post HIV diagnosis challenges that impede coping and adjustment to HIV positive status that, in turn, may shape linkages to and retention in care. Sondela aims to enhance psychological wellbeing and resilience; build gender equitable relationships; prevent intimate partner violence (IPV) and HIV risk behaviour; enhance health seeking behaviour and build social support networks. Specific focus is placed on the attitudes, concerns, and experiences associated with an HIV positive status; sexual and reproductive health and wellbeing; self-care, stress management, positive relationships, as well as the socio-cultural context.

Sondela targets adult heterosexual men and women, immediately after diagnosis or shortly before or after commencing with ART (preferably within 6 months). The design provides for support to PLWH at a time when they may be confronted with the need to acquaint, cope and adjust to news of a positive HIV status thus bridging the gap between the immediate needs of PLWH and the limited psychosocial care facilities beyond post-test counselling services at the health facility level. Sondela was pilot tested among adult men and women over the age of 18 years, but its aims and participatory approaches may be relevant for adolescents living with HIV to provide the much needed health care needs of adolescents [33].

The intervention is named ‘Sondela’ [isiXhosa verb meaning ‘come near’] based on a rationale that the manual is intended to bring people of similar HIV status to connect, share and discuss their experiences in ways that can contribute meaningfully to their common journey of coping and adjusting to positive HIV status. Sondela carries forward the linguistic undertones of being accommodative, non-judgmental and open towards PLWH. Drawing from the Stepping Stones approach originally developed for Uganda [34], and later adapted for South Africa [35], Sondela is based on a theory of change and employs a group-based participatory approach, for delivery by facilitators of the same sex as participants. It is packaged in six 3-hour sessions offered over six consecutive weeks.

## How Sondela was developed

The approach taken to develop Sondela was consistent in principle with the framework proposed by curriculum developers at the UK Medical Research Council who purported that systematic use of the best available evidence, appropriate theory, and piloting the newly developed intervention in a phased manner constitutes best practice [36].

Sondela was developed through an iterative and multi-phased process that ensured a thorough understanding of similar interventions and their impact, and a theoretical understanding of the likely processes of change based on existing evidence and theory [36]. We drew from the findings of a Cochrane review [37, 38], and data from two formative studies that were conducted to explore the needs and coping strategies of HIV positive men [39, 40], and women [41]. Knowledge on barriers to coping and adjusting to new HIV status were also gained from these studies [39–41].

Findings from this work were collated in a coherent and thematic manner and contributed to the development of a logical framework and an evidence informed theory of change. All of these were used to inform the aim, objectives, content of the intervention, determination of target groups and timing of enrolment.

### Systematic review of psychosocial interventions

Two co-authors in this paper conducted the Cochrane systematic review to examine the effectiveness of psychosocial interventions for improving psychological health, psychosocial adjustment and quality of life in adults living with HIV and compared individual versus group based interventions [37, 38]. This review found that there was evidence, albeit relatively low quality by review standards, that psychosocial interventions had significant effects on people’s wellbeing post HIV diagnosis. Yet there were gaps in the type of intervention, the implementation setting, the theoretical approaches, the number of sessions, and the type of facilitators. The majority of the interventions were developed in resource-rich countries with low HIV prevalence rates and different linkages to care concerns relative to low-and-middle income countries. Of the 16 trials reviewed, five targeted homosexual men, and only two targeted women, and so were of uncertain relevance for the predominantly heterosexual and gendered profile of HIV prevalence and linkages to care challenges in South Africa. The theoretical approaches underlying the interventions lacked a gender focus to enable specific relevance to men and women’s needs. Interventions were also longer with an average of 8 to 10 sessions. The majority of facilitators had post graduate or higher levels of education, a resource that is lacking in South Africa. The review recommended the provision of evidence through longitudinal studies evaluating similar interventions in low-and-middle income countries, and the use of standardised measures for outcomes (of psychosocial wellbeing) in all related research. This review begs the need for interventions that reflect the resource capacity in South Africa and account for the gendered needs of PLWH.

### Formative research: Needs and coping strategies of HIV positive adults

As stated, studies to determine needs and coping strategies of men and women contributed to the process of developing Sondela. First, a published study of 18 adult black men living with HIV conducted in Gauteng and Eastern Cape provinces [39, 40], confirmed men living with HIV were less likely to access testing, treatment, or support. This highlighted the importance of psychosocial support and coping interventions for men [39]. Further analysis underscored how constructions of masculinity shape how men deal with living with HIV thus accentuating the gendered experience of living with HIV [40]. Second, an unpublished study among 15 adult black, HIV positive women and 10 service providers in Western Cape province [41] used rapid ethnographic methods usually applied in the design of culturally appropriate research instruments and interventions [42]. The key findings confirmed that following HIV diagnosis, women are faced with difficulties with disclosure, particularly to partners on whom they are financially dependent and whom they fear will abandon or abuse them. Pregnant women were concerned about the well-being of their unborn child. Women also experienced withdrawal from social interaction and community life, and feelings of depression, nihilism and suicide. Similar findings have been observed elsewhere [43]. The two studies demonstrated the gendered risks and social factors that enable or hinder coping and adjustment to HIV positivity. These factors were adapted into the logical framework for the development of Sondela.

### Theory of change

**Fig 1:**
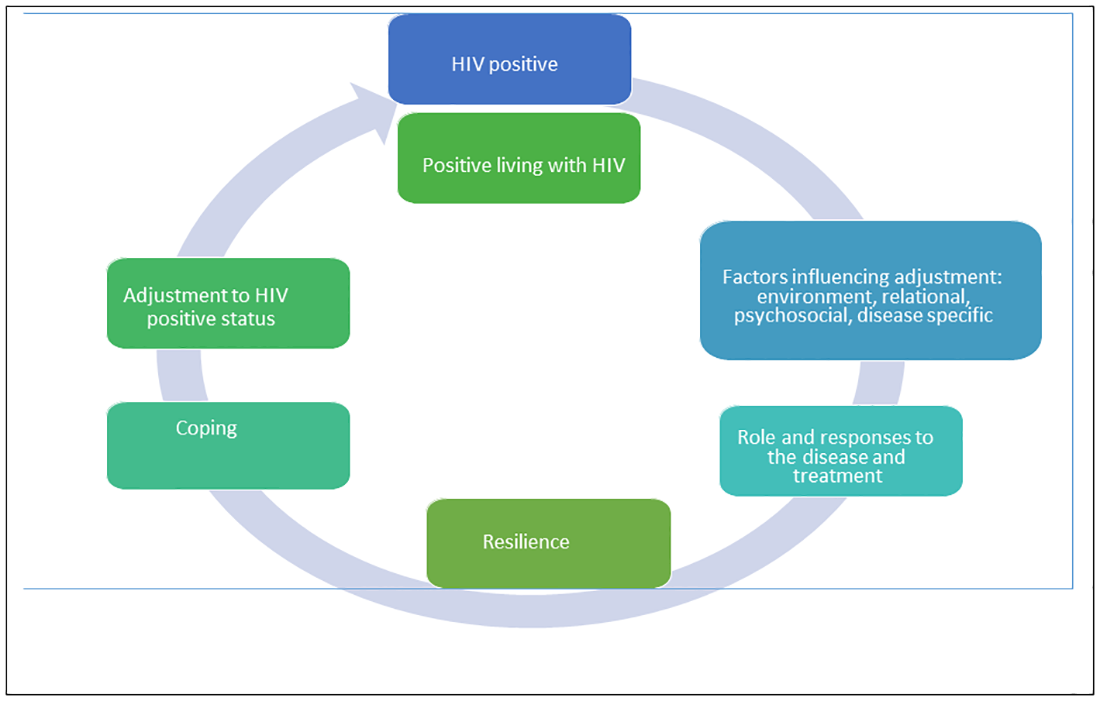
Theory of change.

Sondela views HIV infection as a chronic condition. Our theory of change is based on stress and coping model of psychosocial and physical adjustment to chronic physical illness [44], and the factors contributing to achieving adjustment as an ultimate goal (Fig 1). The assumptions behind Sondela also takes into account the notions of inter-connectedness between environmental, psychosocial, relational and disease-related factors that determine adjustment to chronic illness [45]. Increasing resilience is also critical to ensure one’s ability to overcome adversity and achieve positive outcomes despite challenging circumstances, and helps shifting from positions of vulnerability to resilience [46]. Adjustment, coping, and resilience involve a number of attitudes and behaviours, including constructing realistic life goals, adopting health promoting practices, building self-efficacy and confidence, and maintaining harmonious relationships.

**Fig 2:**
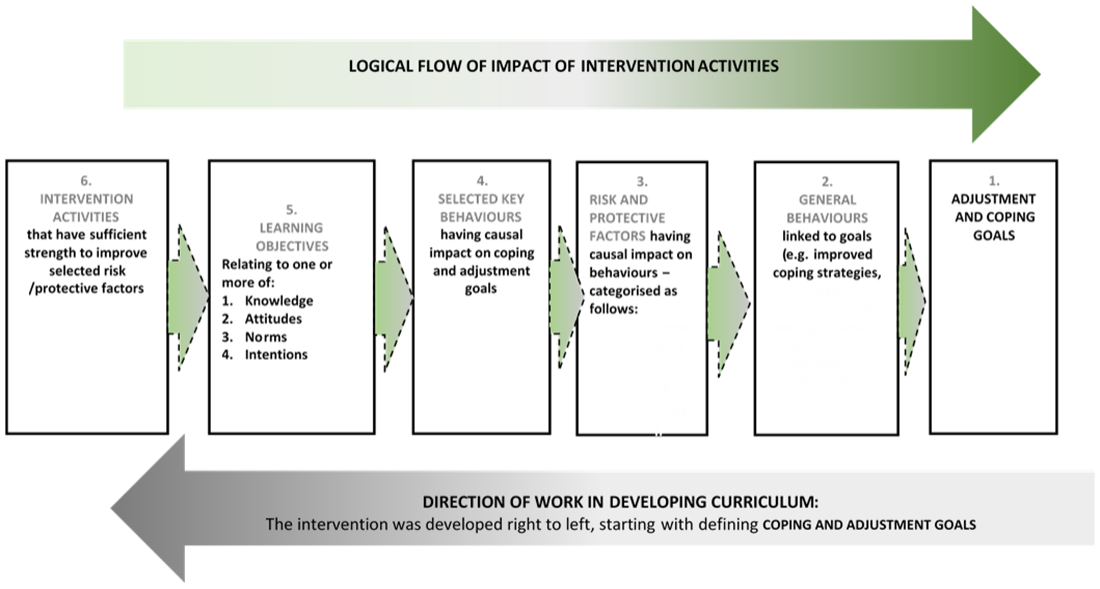
Logical framework for the development of the Sondela intervention.

In line with this theory of change and the attitudes and behaviours associated with adjustment, we outlined a logical framework that took into consideration [47]: (1) the goal of enhancing adjustment to HIV positive status; (2) the behaviours that logically lead to, or impact on adjustment, including a combination of attributes such as coping, resilience, reduced stress, disclosure, harmonious relationships, health seeking, and accessing support systems; (3) risk and protective factors enabling or inhibiting these behaviours; as well as (4) activities that can alter risk and protective factors (Fig 2) [48]. During this process, we identified the risk and protective factors that appeared to influence coping and adjustment to HIV positive status among both men and women and grouped these into five categories: knowledge about HIV, health seeking practices, sexual and reproductive health and rights (SRH) and sexuality, psychological responses to HIV infection, and equitable gender identities.

**Fig 3:**
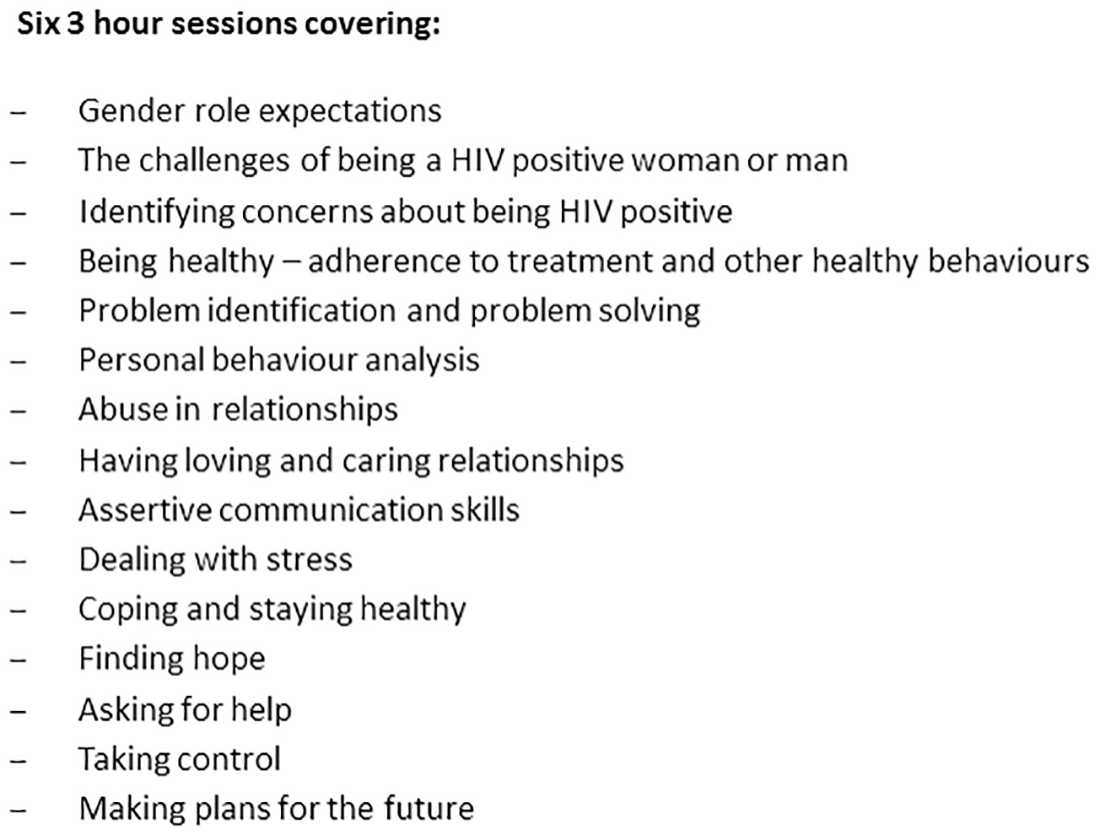
Contents of Sondela.

The identified risk and protective factors were supported by corresponding literature: poor adjustment to HIV positivity is associated with depression, stress and other difficulties with coping [49], which impact negatively on health seeking practices [1, 49, 50]. Depression, stress and difficulties with coping, are the most notable mental health factors, and non-adherence to ART and therapy for opportunistic infections an important health behavioural problem that limits the ability to optimally cope and adjust to HIV positive status [51]. Higher levels of anxiety and hopelessness and lower levels of adaptive responses post HIV diagnosis have similar effects (Kelly et al, 1991). Health and treatment status, social support, substance use, and internalized stigma significantly predict cognitive–affective depression [6]. The effects of accepting one’s HIV positive status as well as the socio-cultural context play a critical role in determining HIV disclosure [52]. There are gender-based differences in men and women’s experiences of living with HIV, including fertility and reproductive health needs [53], sexual risk behaviour [54], and social support [55, 56] and psychological well-being [56]. Among men HIV negative perceptions of HIV and hegemonic conceptions of masculinity hindered men from disclosing their HIV status and seeking health services [57]. Experiences of IPV, fear of stigma and losing a partner impede women’s HIV disclosure [8, 43]. Previous experiences of physical or sexual violence decrease the likelihood of HIV positive women using ART when medically eligible [58, 59]. Consequently, Sondela contains six three-hour sessions that also include a specific focus on prevention of IPV (Fig 3).

Critical reflection is a product of people engaging in discourse and reflection to question themselves and their behaviour and pursue alternative ways of being [60], such that people can appraise and reappraise themselves in ways that improve their life situations. Our aim was for Sondela to encourage the use of critical thinking and reflection as a tool to raise and enhance participants’ internal assessment of various experiences of HIV positivity that influence their behaviour such as gender relations, risky practices and health seeking which could lead to the adoption of new behaviour [60, 61]. Sondela uses motivational therapy as well as a range of well-known participatory techniques such as drama, discussion, and take home written exercises to encourage group discussion, reflection, and critical thinking, used in other group based participatory interventions [48]. Sondela also borrows techniques from problem solving therapy (PST) and a cognitive–behavioural intervention aimed at improving an individual's ability to cope with stressful life experiences [62]. These techniques are designed to help participants to understand their emotions, to view their ‘problems’ more rationally, and to develop effective ways of coping with their stressors. Underlying this intervention are some elements of the social cognitive theory i.e. that behavioural change and the maintenance of behaviour are a function of the expectations about one’s ability to perform a self-efficacy and to achieve the expected outcomes [63, 64].

### Methodology of piloting Sondela

Before rolling out an intervention and conducting a formal impact evaluation, steps need to be taken to gain adequate knowledge about the potential acceptability, relevance and feasibility of the intervention, to ensure the relevance of content, and to address the challenges in the delivery, recruitment and retention of participants into the intervention [36].

#### Recruitment and retention

Men and women living in Gauteng, KwaZulu Natal and Western Cape of South Africa were recruited from public health services to participate in the Sondela workshops. Participants were enrolled if they had been clinically diagnosed with HIV or initiated onto ART within the previous 6 months. HIV counsellors at clinics facilitated recruitment by telling clients who fitted the inclusion criteria about the workshops and inviting them to attend. If clients demonstrated interest, counsellors referred them to the researchers who explained the intervention and obtained their contact details for researchers to inform them of the date, time and place of the workshops. Participants were invited to attend the first session of Sondela and given a choice to discontinue after the first session.

Recruitment suffered some challenges including eligible men and women who feared being stigmatised by group-disclosure when attending the sessions, resulting in a lengthy enrolment period. We also found that among interested participants were those who had been diagnosed for longer. Our strategies to increase recruitment included some of the researchers visiting the health centres, telephone calls to those who expressed interest, and constant updates about our progress. Those who expressed interest but did not turn up or did not complete the programme gave the following reasons: having weekday jobs (at the same time as the workshops), illness, and follow up appointment for CD4 results and treatment. To accommodate those working on weekdays or weekends, we ran Sondela on days that included the weekend. Sondela was delivered in the form of a workshop series which took place over 4–5 days. The pilot allowed us to test not only potential acceptability and relevance of the content but also the form and length of time exercises took. Retention was high as most participants who attended the first session continued with subsequent sessions. Ultimately, a total of 40 men and women participated in pilot of Sondela (see Table 1).

**Table 1:** Distribution of participants by province and by gender.

	Gauteng	Western Cape	KwaZulu Natal	Total
Women	-	12	09	21
Men	08	-	11	19
**Total**				**40**

#### Participants

All participants were Black South Africans with the exception of one male foreign national. The workshops were mainly conducted in the local languages, that is, isiXhosa, isiZulu or Sesotho. They were aged between 18 and 40 years.

#### Process evaluation

Process evaluation of the piloting of Sondela was conducted and involved the assessment of the intervention’s potential acceptability to participants, the relevance of the content, length of exercises and sociocultural setting (see S1 File for some of the questions addressed during the evaluation). During the workshops, the facilitator delivered the sessions and recorded participants’ views and their activities in the workshop, while two colleagues observed the discussions. During each session, the facilitator and observers documented the social attitudes, perspectives and interactions among participants, and the differences and similarities among them. At the end of the final session, a group discussion was held to reflect on the entire Sondela intervention. The discussions allowed access to discourses about the workshop participants’ expectations and experiences in a way they may not necessarily articulate as individuals.

The facilitator and observers’ notes and participants’ feedback from the group discussion were collated and debated, and the facilitator used this information to revise and strengthen the intervention manual. We also conducted thematic analysis from the combined notes from the workshop, observations, and discussions with participants. Themes that emerged from the analysis were discussed among the authors and consensus was reached on the findings and they are presented below.

#### Ethical considerations

This study obtained ethical approval from review committees at the Centres for Disease Control and the South African Medical Research Council. Permission to conduct this study in the clinics was also granted by the provincial Department of Health of the Western Cape, KwaZulu Natal and Gauteng and corresponding municipalities. Participation in the workshops and group discussions was voluntary and written informed consent was obtained from all participants.

## Qualitative feedback

### Programme introduction

Participants appreciated being given an opportunity to choose whether to participate in the intervention. When they attended the first session, participants were invited to make a decision whether to continue attending the workshop. The content of this session covered expectations of an HIV supportive programme, participants’ motivations for participation, how to handle confidentiality in the sessions and the challenges of living with HIV. Participants were given written information about the programme and asked to go home and decide whether to continue with subsequent sessions. Full consent was signed when they returned for the intervention participation and associated research. On average 78% of men and women returned after the first session. This was an initial indication of the acceptability and appropriateness of this approach, as one young man suggested:

The first day was important because it gave us a taste of what we would be missing in other days [if we didn’t attend]. When I got home that day with the little information I got which I never thought would make me feel so free and make me talk… I must say that where I am staying, I have no one that I trust… But when we started talking here and with the little that I was contributing I have realized that I can gain more and share more. And all I can say with this workshop, I have gained because it gave me things that I knew (about) but did not know how to approach (them) and I did not know how to go about them

(P4).

This extract also alludes to issues of trust, confidentiality and HIV stigma that impair disclosure of HIV positive status to family and partners [65], and uptake of HIV testing and ART adherence in South Africa [66, 67]. Fear of gossip about HIV status has potential to dissuade participation in groups that may identify HIV status. We included an exercise that covered trust, confidentiality and non-judgemental attitudes, as well as a confidentiality pledge in Session 1. The confidentiality pledge stated that while the programme facilitators wished for participants to maintain confidentiality of discussions, there was no guarantee that everyone would maintain confidentiality. As such, the onus was on participants to rely on and exercise some goodwill by respecting other people’s information and personal stories that were shared.

The majority of participants completed the intervention and participated in the additional 3 hours of evaluation exercises. This was an indicator of participants’ openness to engage with sensitive topics covered in Sondela. The content of Sondela also covered male and female participants’ fear and feelings of hopelessness related to falling ill, dying, infertility and lack of sexual desire. These sessions allowed participants to express their vulnerability due to HIV positive status. Both male and female participants were concerned about HIV disclosure to partners. Their continued participation seemed motivated by the desire of both men and women to “*understand how to live my life*”, in order to ‘live longer’ with HIV, and mentioned the need to learn how to accept their status. Many participants, particularly men admitted their initial response to their HIV diagnosis was fear of death. Some had tested late as they were beginning to present with opportunistic infections. Men also feared being unable to father children hence their hesitation with condom use. On the other hand, both men and women were unreserved on topics that demonstrated personal conflicts in areas such as multiple concurrent partners and HIV prevention, and reporting multiple concurrent partnerships were more common among men than women.

### Critiquing the benefits of disclosure

HIV disclosure was a great challenge and a source of stress for all participants. Sondela opened discussions about the value and consequences of disclosure, and assisted them in understanding their fears and how to overcome fear in favour of strengthening their psychosocial wellbeing. The challenge with disclosure came from lack of trust of family members, partners, and community members. The workshop environment however, reportedly enabled some participants to surprise themselves as they had not expected themselves “*to be brave to disclose*” to others in the group. They also felt equipped to exercise agency over when and to whom to disclose, contrary to the messages they had received at the health facility which emphasised that “*they needed to disclose regardless of their circumstances*”. One woman realised that “*you can’t disclose until you are ready*, *unlike at the clinic where the message is you must disclose ASAP*”. For others it was “*a big relief to learn about disclosing and that it is my choice when to disclose and who to disclose to*”. In debating the pros and cons of HIV disclosure, some women acted on the knowledge and skills gained and immediately applied their learning. One woman reported that she was “*able to disclose at home during this workshop and the response that I got was good*”.

### Sharing experiences: “We are not the only ones?”

Women and men reported the biggest advantage of participating in the group workshop was an opportunity to meet people like themselves and having a space where they felt comfortable to talk to others about their HIV status. A woman said: “*I was shy but later learned to be free*”. The workshop demonstrated to participants that they were not alone, they were “*not the only ones with issues*, *not the only ones affected by HIV*”. Some reported that it was good to meet with strangers and discuss “*private things*”. One woman had been “*shy*” and had not expected to feel so open in the workshop but as time went, she “*realised [she] had something in common with these women*”. Some women came into the workshop with many questions which they felt were answered, partly because “*if you have something common with the group–it makes it easier to express*”.

For the majority of men, these workshop were the first opportunity to talk openly about their HIV status, feelings about the diagnosis, social and health challenges they were facing as men who live with HIV. Moreover, what men found particularly interesting about the workshops was the interactive approach the facilitator employed, as illustrated in this narrative:

When I attend workshop I expect to be “*workshopped”*, [that] I will sit down and listen to the facilitator talking to me but on this one I was given a chance to participate and contribute ideas. We were formed into a group so that I don’t feel like I am alone. My contribution formed the group thoughts and I related with my group…It gave me a positive idea that getting involved with other people and talk about one common issue that affects all of you helps….

(P1)

Other men concurred with these experiences, as one man reiterated that “*what I thought was a problem for me affects others too*”. Perceived relevance of the content and interest in the workshop was high to the extent that one of the men added his wish that “*this workshop doesn’t stop*”.

### Transformative effects

Participants’ experiences and meanings of concepts were debated, and contradictions and tensions were raised by participants themselves. While Sondela is yet to be evaluated for effectiveness, feedback from men suggests Sondela may have had behavioural transformative effects on men. One man reported his female partner had observed his change since attending the workshop:

…I am happy with my family and the wife is seeing the improvement and she is so happy too. This [intervention] has influenced my life. Every day I think about what I learned in the workshop. Also I would like to appreciate P6 he is so humble

(P8).

Men believed it was more important to first accept their status as HIV positive men and then deal with problems that come with being HIV positive “*patiently*”. They argued for the need for men to take responsibility for their actions and behaviours, and to act and behave responsibly in the first place. The intervention advocated for help-seeking as one of the strategies to support people living with HIV. Male participants supported the idea of asking for help, seeking guidance, and believed doing so was a sign of being ‘*strong’* and ‘*courageous’ [68]*.

### Building harmonious relationships

The Sondela relational exercises promoted building harmonious relationship with partners, family members and friends, and assertive and open communication was a key strategy to ensure this. Both men and women believed that trust and cooperation in intimate relationships were paramount if they were to manage and cope well with their HIV status. Men emphasised expression of love and care towards partners as key to maintaining good relationships, as well as not behaving badly or aggressively towards partners. Some men reported that the stress exercises helped them to reduce the stress of keeping things ‘bottled up inside’. Others valued open communication as a tool to assist them to be less aggressive in conflict resolution, thus addressing some harmful masculinity traits. One male participant said that the “I” statements skills he learned were important because they allowed for solutions, negotiation and ways of talking that are not hurtful. Men reported that they were learning to communicate effectively their likes and dislikes enabling them to take a stance without being physically aggressive.

### “Feeling encouraged”

The relevance of Sondela could also be drawn from the positive effects it had on men’s psychosocial state during the workshop. Initially, some men had described feelings of hopelessness, dejection and suicidal ideation after diagnosis such that it made it difficult for men to talk openly about their HIV status to others.

The men recognised Sondela as providing them the space where they could openly share their problems, participate actively and help one another, as one man indicated:

…Truly speaking men are problematic we do not talk. We close up and when we are diagnosed to be HIV positive we do things that are funny and act like we are losing our minds and yet we are not. This workshop is encouraging us men because we are running away from clinics, we do not want to go there…

Poor relations with family member was seen as a barrier to HIV disclosure, and contributed to men’s fears to communicate problems they had. After attending Sondela as one man stated feeling “*encouraged now [that] I know that there are things I can do as long as I have information*”.

### Feedback on the content and facilitation

The facilitation style and language used also influenced participants’ interest in *Sondela*. Some participants reported that the language that was straightforward yet devoid of shaming or blaming participants. The facilitator addressed sensitive questions that had underlying or direct stigma, mythologies, denialism, or sexually risky behaviour and gender inequitable ideologies, yet simultaneously challenged participants’ attitudes and ideas related to these issues.

Some men had engaged in masculine behaviour associated with increased HIV risk, such as having multiple concurrent partners and binge drinking [69]. Some men engaged in discussions about risk or the unfairness of some gender attitudes without feeling the debates undermined or criticised them.

Women welcomed the sensitive language used to address the mental health consequences of living with HIV and utilising HIV services. They reported their appreciation of intervention perspectives which emphasised people living with HIV are not “diseased”. This expression used by the facilitator crystallised some of the internalised and external stigma women were reporting in the workshop. Women stressed that the facilitator’s statement “you are not the disease, it is the disease that is in you” provided a sense of encouragement and highlighted that they were not to blame for contracting HIV. Some participants reported that they felt they had been labelled in the health facilities, both through the systems of service provision, namely separate queues for HIV positive people, and service providers calling them “*carriers*” of HIV. Women felt that the workshop was different because it treated them with respect and sensitivity. Some men reported their appreciation of working in groups.

Both men and women considered the problem solving exercises to be valuable, and felt more equipped to face problems and resolve them. The comparisons made between their views before and after the workshop demonstrated a high degree of learning from and appreciation of the workshops. One woman explained: “*before the past few days*, *I didn’t know how to manage problems*, *or where to go*, *or how to resolve problems*. *I always focused on my HIV*, *but this support group showed us how to live our lives like everyone else*. *I was in darkness but the group brought me light*. *I now am accepting me and my status*”. These perceptions also influenced their future orientation as one man reported after the workshop: “*being positive doesn’t mean you stop having dreams for the future*, *because you can achieve them*”. This positive outlook generated ideas on how to maintain the new lessons for the future. Women also demonstrated high levels of interest in the intervention, citing its relevance to their health and social challenges, and reiterating the extent to which Sondela helped them to manage their problems. Some considered their problems more manageable compared to before the workshop, and others expressed an overwhelming sense of “*relief*”. Other women reportedly applied the problem solving exercises in other life situations as they felt “*able to help someone else with a problem*, *even if they don’t have HIV*”.

### “Becoming advocates”

The perceived value of Sondela can also be observed from participants’ motivation to share what they learned with others as one woman said: “*I will go out and advise others*”. Some men were motivated to act as agents of change to confront HIV denialism and stigma and prevent the spread of HIV in their communities. They wanted to become active in campaigns, like World AIDS day and 16 Days of Activism and highlighted the prevention of violence against women and children as one of the priority issues for young men to learn ‘ways to act as a good man’. One man was eager to “*teach others the role of being a ‘good man”*. Post-workshop support was important to both men and women, and they discussed strategies to connect with and support fellow participants outside of the workshop. Some men from KwaZulu-Natal did engage in community activism and reported back to one of the co-authors regarding their activities at local schools and the community. They received support from their local clinic to advocate for open communication about HIV, HIV testing, HIV prevention and how to live positively with HIV. However, we were unable to determine how these men conducted these educational activities and whether they sustained them. While at a small scale, these initiatives by some participants demonstrate the potential sustainability of Sondela, where participants are motivated to continue engagements with one another forming supportive relationships beyond the workshop period.

## Effects of critical reflection and participatory approaches

### Adopting a new perspective towards living with HIV

The potentially transformative effects observed among men can be attributed to the participatory methods in Sondela. Men perceived the role plays helped them to learn from their mistakes, and to understand the reality of their problems. Men reported their engagement with other participants with similar social and health challenges influenced their ability to analyse their own behaviour and its consequences to their health and the health of others [70, 71]. Sondela also influenced a shift in men’s perspective about the meaning of HIV in their lives. Men’s perception of themselves as “sick” with HIV was challenged during the workshop. Some began to examine the newly introduced concept that they were not necessarily sick, but ‘just infected’ and were beginning to apply an optimistic view about the meaning of having HIV:

Ja… as a person who is taking ARVs I thought I am sick but what the madam (facilitator) told me is that I am not ‘sick’ just because I am taking ARVs… and [not] allow HIV to bottle me… I am the one who is supposed to contain it and bottle it… So that was a challenge for me to get to understand her [facilitator’s] concept of not being sick while we understood ourselves to be sick… I am literally taking what she taught me and the rest of my life I will not regard myself as sick person

(P5).

### Challenging unhealthy masculine norms

Participatory and critical reflection methods encouraged men to continuously reflect on their current practices and confront gendered behaviours that negatively affected their health–areas of their life that needed to change, as noted by one man that: “*we must have a conscience… We must behave well as HIV positive men*”. After long dialogues about risky sexual behaviour and other harmful masculine behaviour, there was consensus that engaging in multiple sexual relationships or unprotected sex were likely responsible for their having acquired HIV. This is an important finding as it alludes to the potential impact of Sondela on gender norms that justify male sexual entitlement.

Research on harmful masculine practices identified a cluster of male behaviour that increased men’s HIV risk [69]. Men described that a common lifestyle of excessive partying that is often accompanied by harmful intake of alcohol made it likely for men to engage in unprotected sex with one-off or casual sex partners. While they acknowledged this lifestyle posed a risk for HIV, men emphasized these behaviours were difficult to discard. They recognised change was needed urgently and but it had to come from men themselves rather than it be imposed: “*it’s something we must want to change ourselves*”. Critical reflection influenced men to take an objective look at their circumstances and make conscious conclusions about their current harmful behaviour and identify which health promoting practices they should begin to adopt.

Condom use was considered to be a serious challenge, and many men believed that consistency was unachievable. Some men reported that “*condoms are difficult with a main partner and are easier in a one-night stand with other women*”. The logic regarding using condoms at home was about protecting one’s partner regardless of one’s HIV status, but in main partnerships condom use was overridden by the value of trust as has been reported in other studies [72, 73].

Having HIV and the need to use condoms consistently conflicted with men’s desire to father children and superstitions related to maintaining pregnancies. Men considered condoms restrictive of their ability to perform their ‘cultural’ role of “*ukukhulisa umntana”*, a belief that sexual ejaculation is a man’s contribution to nurturing and building body parts of the foetus during pregnancy.

### Challenging unhealthy sexual norms

Among women, discussions on sexuality revealed a greater willingness to use condoms and those who suggested reportedly faced resistance from male partners. As a result, learning negotiation skills to convince male partners to use condoms was a major concern for female participants, and they believed they would have limited success with it. Other women spoke of how sexual relationships had taken a downturn with romance diminishing after HIV diagnosis. For them, having HIV now meant partners were preoccupied with checking whether the condom is in place and showering immediately after sex because of their paranoia about being infected.

### Enhancing ART adherence

Sondela sessions on ART adherence revealed challenges some participants experienced. Adherence on weekends were sometimes challenging for those whose lifestyle involved partying or visiting partners. This resulted in either forgetting or deliberate missing of doses as they did not want to expose their HIV status. One man and one woman were quite remorseful at missing their treatment due to these reasons. The woman said “*I realised my lifestyle can compromise my treatment because I am unable to take it from Friday to Monday [so] I need to have fidelity to treatment*.” Ultimately during feedback sessions, those whose treatment adherence was negatively affected by lifestyle issues realised that they needed to stop clubbing, taking alcohol excessively and needed to be more strategic about ‘hiding’ their medicines from partners and friends while still able to comply [43].

### Participants’ goal setting and future plans

Coping and management of stress is associated with positive future orientation [74]. Women were more practical about their life needs, and described how they would pursue a range of goals and aspirations in order to ensure they could maintain a good state of wellbeing after the workshop. Women were quite motivated to compete their schooling and get a job, as one woman claimed that “I know what I want”. Others wanted to maintain their health by reducing risky behaviours, such as non-condom use, and by ending abuse in their relationships, but many believed getting jobs was one of the ways to take better control of their lives and enhance their wellbeing. Many women had adequate knowledge and information about HIV before attending the group, but felt Sondela taught them in their real life contexts which was relevant to their lives. They learnt more about ‘life’ than ‘HIV’, as one woman explained:

The topics were relevant, that you can talk about HIV and how to manage stress—because we have lots of stress. And to learn about abuse—that happens in our community, the role plays we did show us how men beat us and now we know how to manage it.

Sondela also addressed relevant issues on gender inequities in women’s relationships. Women described experiences of intimate partner violence, including pressure to have sex without condoms and their own inability to negotiate condom use. Such experiences contributed to some women’s psychosocial distress as one woman indicated: “*he will be saying you gave him HIV*, *and blame you*, *yet you know he had if from the start*”. Some women reported that “*he insults me about my HIV whenever we argue*”. The effects of such conflict also contributed to women’s low self-esteem such that some perceived themselves as “*stupid*” and “*easily manipulated*”. This had disempowering effects on some women who believed they merely “*persevered*” in their relationships and had no power to change them. Evidence of critical reflection appeared when there was consensus among women about their role in being complicit with ideologies that support male gender power at the expense of themselves. These kinds of discussions were quite tough for some women, as they began to reflect on how their ideas and values perpetuated some of the challenges that increase inequitable gender relations. The communication exercises, however, were found to be useful in equipping women to assert themselves in similar situations. Some women found sharing the content of the workshop with partners brought them closer and gave them confidence to talk more openly in the future. Another woman claimed that: “*I am feeling free because I can now talk to my partner about things we couldn’t/didn’t talk about before*”.

## Conclusion and recommendations

The pilot testing of Sondela with men and women has shown a high level of interest in and relevance for the participants. The need for such interventions was a recurring theme from both men and women living with HIV. This is particularly relevant after the announcement by the South African Minister of Health that Government decision to adopt the WHO Test and Treat HIV (TnT) policy with scale up commencing in September 2016 with sex workers, as one of the key high-risk populations prioritised in the National Strategic Plan to Eliminate HIV, STIs and TB in South Africa.

Scaling up the TnT approach to HIV populations in South Africa has potential to curb HIV transmission, provided there is wide-scale coverage of HIV testing, immediate initiation and high adherence to ART as well as efficient case management. This implies the increased need for prompt linkage and sustained retention in HIV medical care after diagnosis [19]. To achieve this, TnT should strengthen linkages to and retention in HIV care through the provision of crucial supportive services, particularly, psychosocial support mental health services [75, 76]. Sondela has the potential to bridge the gap in addressing psychosocial challenges facing this population. TnT requires adherence for it to be effective; consequently, the health system will need to devise effective interventions to curb non-adherence. Sondela contributes to curbing the lack of coping strategies which are associated with non-adherence to ART as observed among male participants who sought to ‘become advocates’ of acceptance of HIV positive status in order to encourage men to seek wellness. There is an opportunity for Sondela to contribute as one of the programmes to help to enhance the effectiveness of the broader TnT programme.

Sondela is flexible in its implementation to attract difficult to reach groups including those who have limited access to day-time HIV services. This highlighting the potential replicability of the intervention in other settings and can be ascertained through rigorous evaluation in a research environment. Rigorous evaluation will determine not only its acceptability to a wider target population or feasibility in other settings, but also its effectiveness in improving adjustment to HIV status and ultimately linkages to and retention to HIV care. Scientific evaluation that is closely linked with broader HIV care services such as CD4 testing at diagnosis and ART administration may assist in the quality improvement of monitoring and evaluation of the HIV care and treatment cascade, and in maintaining primary care level contact with recently diagnosed populations thus maximising linkages to and retention to HIV care particularly among men and other high risk groups.

## Supporting information

S1 File Evaluation discussion with intervention participants. (PDF)
