# Supplementary material for: "I was in the darkness but the group brought me light": Development, relevance and feasibility of the Sondela HIV adjustment and coping intervention
Source: PLoS One. 2017 Jun 1;12(6):e0178135. doi: 10.1371/journal.pone.0178135 (PMC5453489; doi:10.1371/journal.pone.0178135)
Supplement: S1 File — (PDF) [file pone.0178135.s001.pdf]

S1 File: Evaluation discussion with intervention participants

Appropriateness of content and materials:

- What are the needs of HIV positive people attending these sessions?
- What were your expectations when you first joined the group?
- So far, do you think the group has managed to meet these expectations? If YES, how so? If NO, why not?
- Tell me about the topic of the session – was it relevant to your lives?
- What about the activities – were they relevant to the topic? How are these activities helpful to you?
- Were the materials easy to understand? What do you remember most about the activities in the session? Which do you remember the most? What materials or content was difficult/embarrassing? Was any part not relevant for your community?

Gender:

- How did the support group meet the specific needs of men/women?

Overall satisfaction with intervention:

- What did you like about the session/intervention? What didn't you like?
- What will you remember most about the session/intervention?
- How did the session/intervention make you think and see things differently?

Group environment:

- Please share with me your positive experiences of being a member of this group?
- Did you receive support from the group? What did this entail?
- Describe the trust in the group?
- Did you develop good friendships in the group?
- Describe the negative experiences of being a member of this group?
- Would advise another HIV positive person to join a group like this? Yes or No, please explain.

Facilitation:

- Was the facilitator well prepared for the session/intervention?

Attendance:

- What are some ways to get more HIV positives to attend interventions?
- Why would HIV positives not attend such sessions? What would improve the intervention?
